# New mitochondrial genomes of three whip spider species from the Amazon (Arachnida, Amblypygi) with phylogenetic relationships and comparative analysis

**DOI:** 10.1038/s41598-024-77525-0

**Published:** 2024-11-01

**Authors:** Acácio Freitas Nogueira, Eder S. Pires, Guilherme Oliveira, Leonardo Carreira Trevelin, Santelmo Vasconcelos

**Affiliations:** Instituto Tecnológico Vale, Rua Boaventura da Silva 955, Belém, Pará CEP 66055-090 Brazil

**Keywords:** *Charinus*, *Heterophrynus*, Ka/Ks ratio, Cave species, Mitochondrial genome, Phylogenetics

## Abstract

The complete mitochondrial genomes of the whip spiders *Charinus carajas*, *C*. *ferreus*, and *Heterophrynus longicornis* were sequenced, annotated, and compared with other mitogenomes of whip spiders and arachnids. The three new mitogenomes have the 37 genes usually observed in Metazoa: 13 protein-coding genes (PCGs), 22 transfer RNAs (tRNAs), and two ribosomal RNAs (rRNAs), plus a non-coding control region (CR). Most PCGs presented an ATN start codon, except *cox1* in both *Charinus* species, initiating with TTA. Most PCGs terminated with stop codons TAA or TAG, except *nad5* of *C. carajas* and *cox3* of *H. longicornis*, which presented an incomplete stop codon (T). The Ka/Ks ratios were less than one for all the PCGs, indicating these genes are under purifying selection. All the tRNAs, except for serine 1 (*trnS1*), had the typical cloverleaf-shaped secondary structure. All the phylogenetic analyses resolved *Charinus carajas* and *C*. *ferreus* as monophyletic groups. Nonetheless, we did not recover the monophyly of *Heterophrynus longicornis*. The phylogenies under partitioned models did not recover suprageneric taxonomic groups as clades, but the Bayesian inference under the CAT infinite mixture model recovered the family Phrynidae and the superfamily Phrynoidea as monophyletic groups.

**Supplementary Information:**

The online version contains supplementary material available at 10.1038/s41598-024-77525-0.

## Introduction

The arachnid order Amblypygi, the whip spiders, are unique organisms with dorsoventrally compressed bodies, long and raptorial pedipalps, and an extremely elongated antenniform first pair of legs that are not used for locomotion but as specialized sensory structures^1^. Despite being a relatively small order with approximately 278 living species^2^, whip spiders are found in tropical and subtropical regions worldwide^3,4^, with the most diversity hosted in South America^5^. Systematics and phylogenetic relationships in Amblypygi have been investigated mostly using morphologic characters^6–9^, however, recent studies have also used molecular data to infer phylogenetic relationships in the order^4,10^. In early cladistics studies and recent molecular phylogenetic analyses, Amblypygi has been resolved as a monophyletic group^6,10–13^. Another undisputed relationship is Amblypygi as the sister group to Uropygi, a clade formed by the orders Thelyphonida (whip scorpion) and Schizomida (short-tailed whip scorpions), with these three orders forming the clade referred as Pedipalpi^12–15^.

Molecular data have also been employed in studies of cryptic diversification and phylogeography of whip spiders^16–20^. In this trend, complete mitochondrial genomes (mitogenomes) have been used to infer phylogenetic relationships in Arachnida^21,22^. However, complete mitogenomes of only two whip spiders, *Damon diadema* and *Phrynus* sp., have been published so far^21^, which hamper the use of most of these organisms in mitogenomic studies of biodiversity.

Therefore, given the lack of available mitochondrial genomes for other Amblypygi taxa, we sequenced and annotated the complete mitochondrial genomes of three species occurring in the Eastern Amazon, *Charinus carajas*, *C*. *ferreus* and *Heterophrynus longicornis*. *Charinus* was the type genus of the pantropical family Charinidae^8^. However, recent changes in the classification of Amblypygi based on a global molecular phylogeny using ultraconserved elements (UCEs), as well as the combination of UCEs with six Sanger loci, have synonymized Charinidae with Charontidae. *Charinus* now belongs to Charontidae, the most diverse family in the order, with around 150 species^10^. The two sequenced *Charinus* are narrow-range species endemic to ferruginous caves in the Serra dos Carajás^5^, an iron ore mining region, while *H. longicornis* is widely distributed in northern and central South America^16^. This study aims to determine the features of these *de novo* assembled mitochondrial genomes, to compare the data of the three species with those of other related arachnids (including other Amblypygi taxa), and to elucidate the phylogenetic relationships of the herein sequenced species with other whip spiders based on mitochondrial sequences.

Furthermore, mtDNA has been one of the most popular markers for the study of molecular biodiversity in animals^23^. The reasons for that are specific biological properties, such as the high variability of mitochondrial sequences in natural populations because of its elevated mutation rate^24^, which can generate some signals about population and species history over short time frames^23^. In addition, the clonal inheritance of its whole genome, which behaves as a single non-recombining locus, makes it an outstanding marker for tracing the evolution of genealogies^25^. These properties combine elevated polymorphism and traceability, indispensable features for classical phylogeography^25^, species identification^26^, and modern biodiversity monitoring via eDNA metabarcoding^27^.

## Results and discussion

### Overall genomes features

**Fig. 1:**
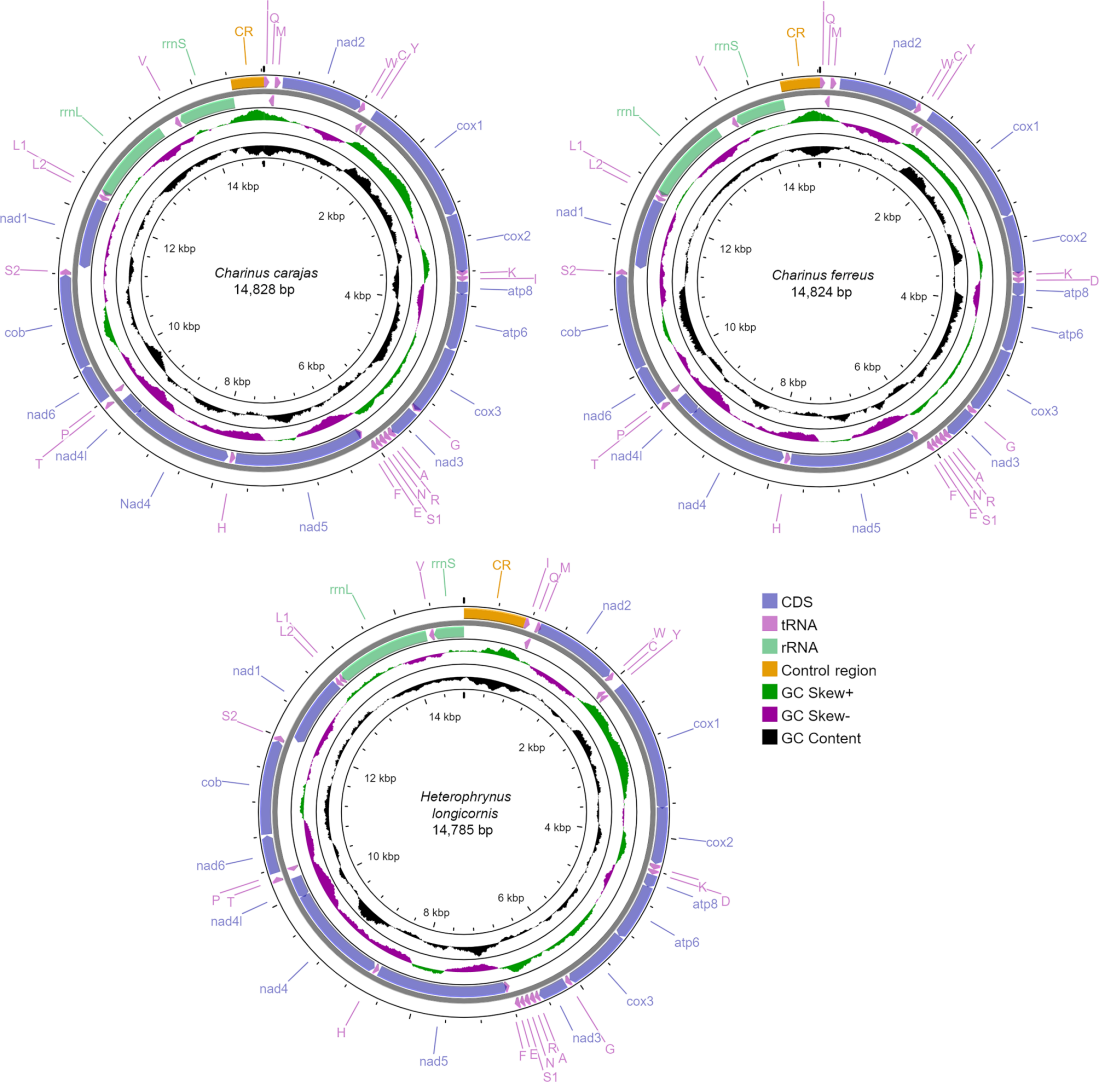
Circular maps of the complete mitochondrial genomes of *Charinus carajas*, *C*. *ferreus*, and *Heterophrynus longicornis*, showing the PCGs (CDS), rRNAs, tRNAs, and the control region. The images were generated using the CGView/Proksee online server (https://proksee.ca/)

The complete mitochondrial genomes presented lengths of 14,785 bp for *Heterophrynus longicornis*, 14,828 bp for *Charinus carajas*, and 14,824 bp (two samples) or 14,822 bp (one sample) for *Charinus ferreus* (Fig. 1). The pairwise identity among samples of *C*. *carajas* was 99.8%, and 99.9% among samples of *C*. *ferreus*. Nucleotide composition was approximately 35% of A, 28.9% of C, 8.9% of G, and 27.2% of T for *C*. *carajas*; 32.9% of A, 34.4% of C, 9.5% of G, and 23.2% of T for *C*. *ferreus*; and 32.6% of A, 28% of C, 9.7% of G, and 29.7% of T for *H*. *longicornis*. All the mitogenomes were positively skewed for the AT proportion (0.125 for *C*. *carajas*; 0.173 for *C*. *ferreus*, and 0.047 for *H*. *longicornis*), and negatively skewed for the GC content (− 0.529 for *C*. *carajas*; −  0.567 for *C*. *ferreus* and − 0.485 for *H*. *longicornis*). These considerably high negative values of GC skew were also observed in the mitogenomes of the two previously sequenced Amblypygi (− 0.473 for *Damon diadema* and − 0.453 for *Phrynus* sp.), being yet negative in the mitogenomes of the whip scorpion *Mastigoproctus giganteus* (−  0.297)^21^ and the Schizomida *Schizomus zhensis* (−  0.393)^28^. However, the known Araneae mitogenomes reported positively skewed GC content, as in the cases of *Nephila pilipes* (0.240), *Trichonephila antipodiana* (0.271), *T*. *vitiana* (0.266)^29^, and *Cheiracanthium triviale* (0.194)^30^. Considering the phylogenetic relationships of the cited taxa, with Uropygi being the sister group to Amblypygi and both forming Pedipalpi, the clade sister to Araneae, these results may indicate a bias toward the excessive use of Cs over Gs from Araneae to Amblypygi or vice versa. AT skewness presents a less notable pattern because all the amblypygids herein sequenced plus *Damon diadema* (0.101) exhibited positive values, whereas *Phrynus* sp. was the only exception with a value of −  0.03^21^. On the other arachnids, all the Araneae and Thelyphonida species analyzed presented negative values of AT skew^21,29,30^, but *Schizomus zhensis* presented positive (0.084) AT skew.

The mitogenomes assembled here contained the typical 37 genes of bilaterians, being 13 protein-coding genes, 22 transfer RNAs, and two ribosomal RNAs (*rrnL* and *rrnS*), as well as a putative control region characterized by a non-coding A-T rich segment (Fig. 1; Table 1). The gene arrangement in the three mitogenomes is identical to those of *Damon diadema* and *Phrynus* sp. Such gene order is almost equal to that of Pycnogonida mitogenome (from which differs by a single tRNA translocation event), which was inferred to represent the putative ancestral gene order of Chelicerata based on an analysis of 464 complete arthropod mitochondrial genomes^31^. Twenty-three genes (nine PCGs and 14 tRNAs) are encoded on the majority strand (J-strand) and the other 14 (4 PCGs, 8 tRNAs, and 2 rRNAs) are encoded on the minority strand (N-strand). The mitogenome of *C*. *carajas* contained 14 genic overlaps, ranging from one to 45 bp, while *C*. *ferreus* presented 15 overlaps, ranging from one to 20 bp, and *H. longicornis* with nine overlaps, ranging from one to 17 bp. The number of intergenic spacers, excluding the control region, was 13 in *C*. *carajas* and *C*. *ferreus*, and 16 in *H*. *longicornis*. The longest intergenic spacer in all species was between *rrnL* and *trnV*. Although this interval had reached a few over 200 bp in both species of *Charinus*, it was just 35 bp in *Heterophrynus* (Table 1).

**Table 1:** List of annotated genes and their features in the mitochondrial genomes of *Charinus carajas* (C), *Charinus ferreus* (Cf), and *Heterophrynus longicornis* (Hl)

Gene	Strand	Length			gen. ovl./inter. spac.			Start/stop codons		
		Cc	Cf	Hl	Cc	Cf	Hl	Cc	Cf	Hl
*trnT(tgt)*	+	60	63	63	0	0	0			
*trnP(tgg)*	−	63	65	62	5	3	− 15			
*nad6*	+	432	432	459	− 1	− 1	13	ATT/TAA	ATC/TAA	ATA/TAA
*cob*	+	1131	1137	1128	− 2	− 2	− 2	ATG/TAA	ATG/TAA	ATG/TAG
*trnS2(tga)*	+	70	66	68	21	19	6			
*nad1*	−	915	915	915	4	4	2	ATG/TAA	ATG/TAA	ATG/TAG
*trnL2(taa)*	−	68	66	63	2	3	1			
*trnL1(tag)*	−	66	65	63	− 45	0	0			
*rrnL*	−	1118	1083	1226	202	201	35			
*trnV(tac)*	−	70	67	66	9	1	− 3			
*rrnS*	−	749	665	395	0	0	0			
*CR*		389	469	731	0	0	0			
*trnI(gat)*	+	66	64	65	− 3	− 3	9			
*trnQ(ttg)*	−	69	66	67	− 1	− 1	3			
*trnM(cat)*	+	70	70	65	0	0	0			
*nad2*	+	996	996	984	− 2	− 2	− 2	ATC/TAA	ATA/TAA	ATC/TAA
*trnW(tca)*	+	67	67	62	− 1	− 1	0			
*trnC(gca)*	−	62	65	63	1	3	0			
*trnY(gta)*	−	61	63	60	− 5	− 5	− 8			
*cox1*	+	1536	1536	1560	3	3	− 17	TTA/TAA	TTA/TAA	ATC/TAA
*cox2*	+	670	670	681	0	0	1	ATG/T(AA)	ATG/T(AA)	ATG/TAA
*trnK(ctt)*	+	68	69	68	− 1	− 1	0			
*trnD(gtc)*	+	66	68	62	0	0	1			
*atp8*	+	156	156	156	− 7	− 7	− 7	ATC/TAA	ATC/TAA	ATG/TAA
*atp6*	+	672	672	681	3	5	− 1	ATG/TAA	ATG/TAA	ATG/TAA
*cox3*	+	785	786	784	− 1	− 1	0	ATG/TA(A)	ATG/TAA	ATG/T(AA)
*trnG(tcc)*	+	67	68	63	0	0	6			
*nad3*	+	346	348	342	0	− 2	0	ATT/T(AA)	ATC/TAG	ATT/TAG
*trnA(tgc)*	+	70	67	56	0	− 1	1			
*trnR(tcg)*	+	64	63	64	− 1	− 1	2			
*trnN(gtt)*	+	66	64	64	0	0	0			
*trnS1(gct)*	+	55	55	56	1	1	1			
*trnE(ttc)*	+	64	63	62	− 4	− 4	4			
*trnF(gaa)*	−	67	68	63	0	− 1	0			
*nad5*	−	1660	1668	1699	4	4	0	ATT/T(AA)	ATT/TAG	ATG/T(AA)
*trnH(gtg)*	−	70	74	62	26	16	3			
*nad4*	−	1332	1329	1338	− 20	− 20	− 20	ATG/TAA	ATG/TAA	ATG/TAG
*nad4l*	−	282	282	285	23	23	21	ATG/TAG	ATG/TAG	ATG/TAG

CR = control region; length in bp; minus sign indicates genic overlaps (gen. ovl); positive numbers indicate the number of bases as intergenic spacers (inter. Spac.). 3’ a residues added to the mRNA to complete the stop codons are put between parentheses

### Protein-coding genes and codon usage

The complete extension of the coding regions was 10,913 bp for *C. carajas*, 10,897 bp for *C*. *ferreus*, and 11,012 bp for *H. longicornis*. The total length of each PCG in the mitogenomes of each species, as well as their initial and terminal codons, are listed in Table 1. All the PCGs initiate by the usual start codon ATN (N = A/T/G/C), except for *cox1* of *Charinus*, which initiate with the unusual codon TTA, as also observed for *Phrynus* sp. and other arachnids (namely, *Nephila pilipes*, *Trichonephila antipodiana* and *T*. *vitiana*)^21,29^. Nevertheless, *Cheiracanthium triviale*, another Araneomorphae species, has unusual start codons (TTA, TTG and TTT) in other PCGs but not in *cox1*^30^. Most PCGs terminate with one of the complete stop codons TAA or TAG, except *cox2* of both *Charinus* species, *cox3* of *C*. *carajas* and *H*. *longicornis*, *nad3* of *C*. *carajas*, and *nad5* of *C. carajas* and *H*. *longicornis*, which terminate with an incomplete stop codon T or TA. Truncated stop codons consisting only of a T or a TA were also observed in *Phrynus* (three genes) and *Damon* (five genes) mitogenomes, and such incomplete stop codons are presumed to be completed by post-transcriptional polyadenylation^32^.

The average A + T content was 61.0% for *H. longicornis* and *C*. *carajas*, and 52.5% for *C*. *ferreus*, ranging from 59.8% (*cox3*) to 64.0% (*nad3*), from 57.3% (*cox1*) to 67.9% (*atp8*), and from 51% (*nad3*) to 57.4% (*nad4l*), respectively. Leucine1 was the most frequently used amino acid (AA) in both *Charinus* mitogenomes, while Isoleucine was the most observed in *H. longicornis*. Cysteine was the least used AA in the three mitogenomes. The RSCU for all AAs in the three species are shown in Supplementary Figure S1.

**Fig. 2:**
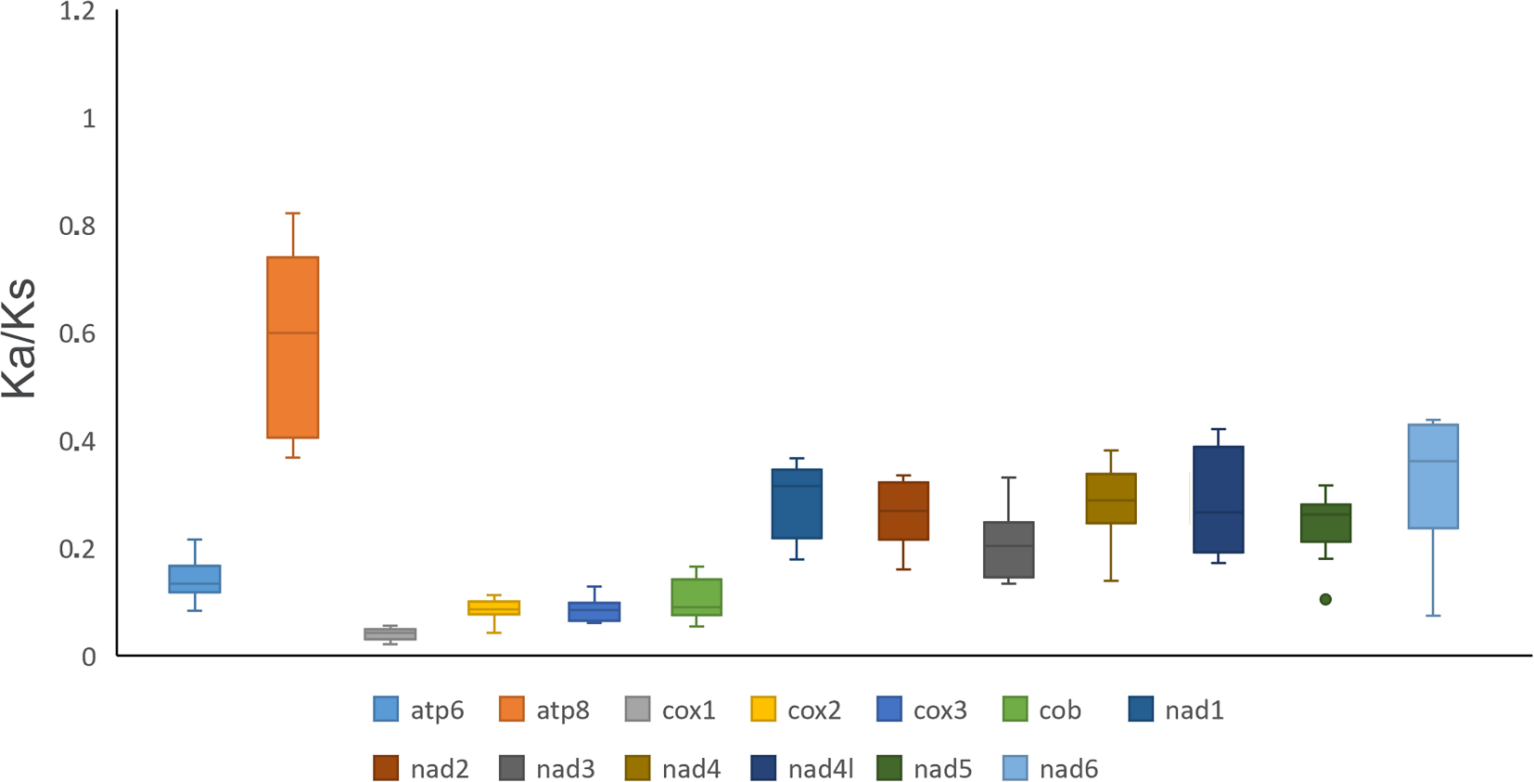
Box plot for pairwise divergence of Ka/Ks ratio of the 13 PCGs in the mitogenomes of Amblypygi (*n* = 5: *Charinus carajas*, *C*. *ferreus*; *Damon diadema*, *Heterophrynus longicornis* and *Phrynus* sp.). The analysis was computed in DnaSP v6

The Ka/Ks ratios were less than one for the 13 PCGs (Fig. 2), indicating that all these genes are under purifying selection. The *cox1* and *atp8* genes had the lowest and the highest Ka/Ks ratios, respectively, as previously reported for spiders^29,33^. Following an increasing order we found: *cox1* < *cox3* < *cox2* < *cob* < *atp6* < *nad3* < *nad5* < *nad2* < *nad4* < *nad4l* < *nad1* < *nad6* < *atp8*. In addition, the fact that *cox1* presented the lowest Ka/Ks ratio in the mitogenomes of whip spiders, representing fewer changes in amino acids, gives support to its use as a suitable molecular marker for DNA-based species delimitation within the order, as well as reported in other arachnids^34,35^.

### Transfer and ribosomal RNAs

The size of the tRNAs ranged between 56 and 68 bp in *H. longicornis*, 55–70 bp in *C. carajas*, and 55–74 bp in *C*. *ferreus* (Table 1). The sum of all the sequences of tRNAs had lengths of 1387 bp in *H*. *longicornis*, 1449 bp in *C. carajas*, and 1,446 bp in *C*. *ferreus*. In the *Charinus* species, all the transfer RNAs except the tRNA of serine1 (*trnS1*), have the typical cloverleaf-shaped secondary structure. The predicted secondary structures of all tRNAs of the three species are shown in Supplementary Figures S2-S4. The *trnS1* lacks the DHU arm, which is not uncommon since this seems to be a feature broadly shared among metazoans, with the metazoan ancestor losing the DHU arm structure before its diversification^36^. The mitogenome of *H. longicornis* differs from those of *Damon* and *Phrynus*, the more related genera, by lacking DHU and TΨC arms in the *trnA*, while in *Damon diadema* and *Phrynus* sp., this tRNA only lacks the DHU arm^21^. In contrast, many mitochondrial tRNAs of spiders have atypical or aberrant cloverleaf secondary structures, lacking DHU and TΨC arms^29,33,37^.

The location of the two ribosomal RNA genes is the same in *Charinus* and *Heterophrynus*: *rrnS* is located between *trnV* and the control region and *rrnL* between *trnL1* and *trnV*. The position of these rRNA genes, relative to the annotated regions of the genome, is the same found in the other whip spiders, *Damon* and *Phrynus*, and in Thelyphonida and Schizomida, in accordance with Amblypygi and Uropygi as closely related taxa forming the clade Pedipaldi^12^. In contrast, in araneid spiders, *rrnL* is located between the same genes, but *rrnS* is positioned between *trnV* and *trnQ*^29^. This difference is probably due to changes in the position of *trnQ* since tRNAs are more prone to rearrangement than the PCGs and rRNAs^22^.

### Control region

In all mitogenomes, the non-coding control region (CR) was located between *rrnS* and *trnI*, as found in the other known mitogenomes of whip spiders. The base content is A + T rich in the three species, being 69.6% in *H*. *longicornis*, 68.9% in *C*. *carajas*, and 65.2% in *C*. *ferreus*. AT skewness was slightly negative for *H*. *longicornis* (−  0.037) and *C*. *carajas* (−  0.016), contrasting with the positive values discovered for the other regions and the whole mitogenomes, and positive for *C*. *ferreus* (0.098). GC skewness was negative for all the species (−  0.395 in *H*. *longicornis*, −  0.209 in *C*. *carajas*, and − 0.239 in *C*. *ferreus*).

### Phylogenetic analyses

**Fig. 3:**
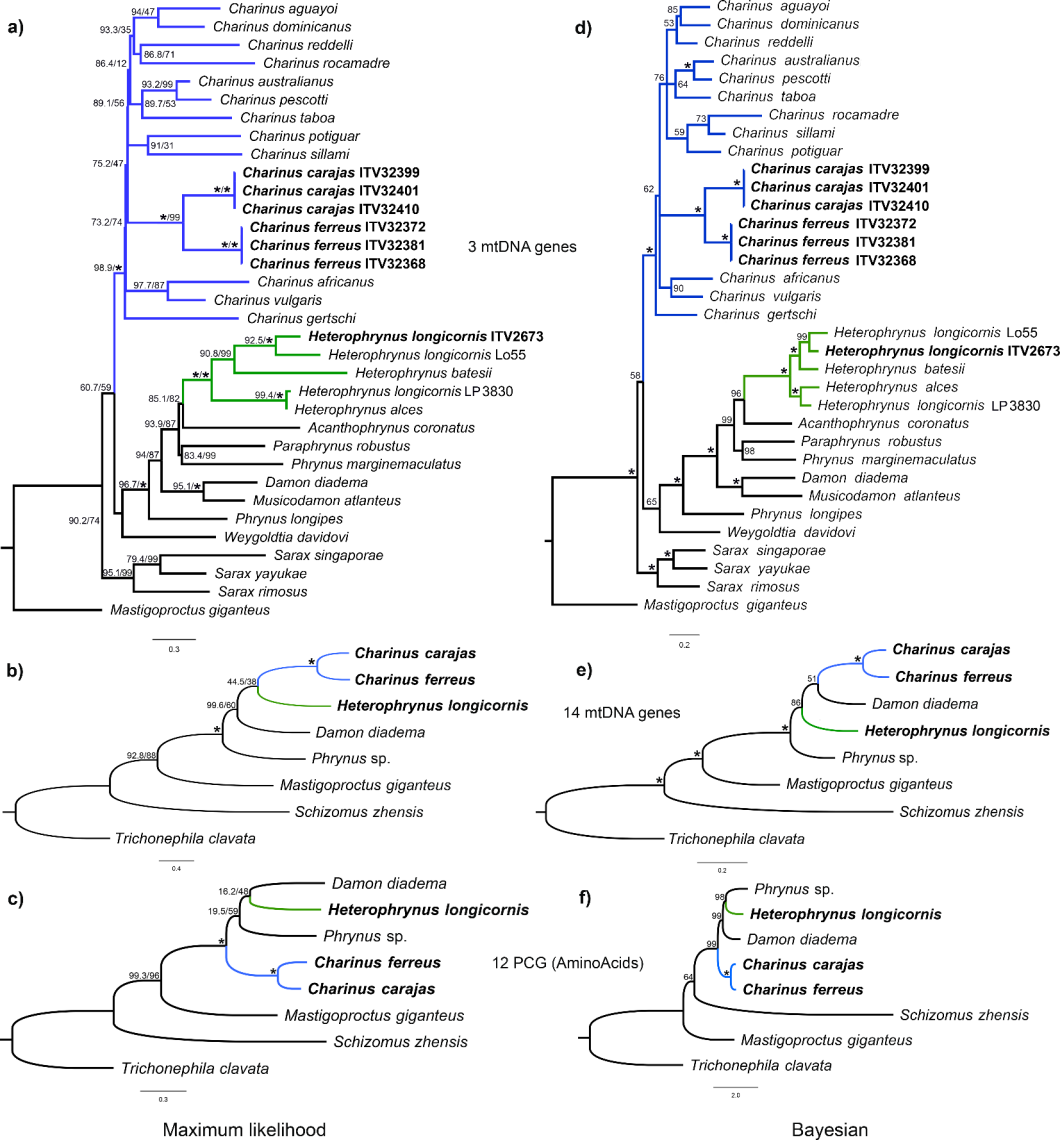
Phylogenetic trees showing the relationship among the species herein sequenced with other arachnids using maximum likelihood (ML) (**a**–**c**) and Bayesian analyses (**d**–**f**). Branches in blue represent the genus *Charinus*; branches in green represent the genus *Heterophrynus*. Numbers on nodes are SH-aLRT/UFBoot supports in ML analyses and posterior probabilities (PP) in percentage in Bayesian analyses; asterisks indicate full node supports (i.e., SH-aLRT, UFBoot and PP = 100). Boldface terminals indicate novel mitogenomic data from this study. The three-gene matrix (**a**, **d**) with only *cox1* and rRNA sequences has a more extensive taxonomic sampling. The complete datasets, with the mitogenomes of *Damon diadema*, *Phrynus* sp., *Mastigoproctus giganteus*, *Schizomus zhensis*, and *Trichonephila clavata*, used nucleotide sequences of 14 genes (12 PCGs and rRNAs) (**b**, **e**) or amino acid sequences of the 12 PCGs (**c**, **f**). The monophyly of Phrynidae and Phrynoidea was only obtained in the PhyloBayes tree under the CAT-GTR model on amino acids (**f**).

In the taxonomically more comprehensive phylogenetic trees of the three-gene matrix (Fig. 3a, d), *Charinus carajas* and *C*. *ferreus* only grouped with conspecifics, recovering both as monophyletic groups. The two species were also recovered as sister clades. These relationships were recovered in Bayesian and maximum likelihood (ML) trees with high supports (see Fig. 3a, d), indicating these species are well delimited from each other. Our sequence of *Heterophrynus longicornis* clustered with the other congeneric species, assigning it to this genus with maximum clade support (UFBoot = 100, and percentages of posterior probabilities (PP) = 100). However, the three specimens ascribed to *H*. *longicornis* did not form a clade. Our sample (ITV2673), from the eastern Amazon (Brazil), recovered as sister to the exemplar Lo55 (Hl-B-CdP1-Lo55, BIN: ADC2132), from northeastern Brazil in the Caatinga-Cerrado biome (A Savana-kind ecosystem), whereas the exemplar LP 3830 (AMCC LP 3830), from French Guiana (northern Amazon), retrieved as sister to *H*. *alces* (Ha-FG-Jose-A7), also from the French Guiana. The exemplar of *H*. *batesii* came from the southwestern Amazon and was recovered as the sister to the two *H*. *longicornis* from Brazil. Therefore, all *Heterophrynus* samples from Brazil, irrespective of the taxon assigned, formed a monophyletic group, and the same happened with the specimens from French Guiana. Species of *Heterophrynus* are widely distributed throughout central South America and may occur in sympatry^16,38^. They can exhibit a high level of intraspecific mtDNA genetic divergence, which accounts for the cryptic diversity in *H*. *batesii* and *H*. *longicornis*^16^. Thus, the finding of a phylogenetic relationship shaped by geography, unexpected according to taxonomy, indicates that cryptic diversity in the genus needs to be disentangled before any accurate systematic conclusion can be reached. Such a proposal would demand sampling more species and a systematic revision, which is beyond the scope of this paper.

In the maximum likelihood (ML) and Bayesian trees of the nucleotide matrix of 14 genes (Fig. 3b, e), the two *Charinus* species formed a clade with maximum node support (UFBoot = 100, PP = 100). *Heterophrynus longicornis* was sister to the *Charinus* clade in the ML tree and to a clade formed by *Damon diadema* and *Charinus* in the Bayesian tree. However, these relationships were not significant (UFBoot and PP < 95) in both trees. Those topologies did not recover Phrynidae (*Heterophrynus* and *Phrynus*) or Phrynoidea (*Damon*, *Heterophrynus* and *Phrynus*) as monophyletic groups.

In the ML tree of the amino acid (AA) matrix (Fig. 3c), *H. longicornis* was sister to *Damon*, and the two formed a clade with *Phrynus*, thus gathering only all members of Phrynoidea into a monophyletic group, even though Phrynidae has not yet been recovered. Again, the statistical support for those relationships was not significant. Only in the Bayesian tree of the AA matrix (Fig. 3f) did *Heterophrynus* and *Phrynus* arise as sister taxa. *Damon* was recovered as the sister to the clade formed by those two species. Moreover, these relationships were highly supported. Thus, the monophyly of the family Phrynidae and the superfamily Phrynoidea were only recovered in the Bayesian inference from amino acids, in which we implement the CAT mixture model.

In the phylogenomics of Amblypygi, all datasets (UCE matrices and their combination with Sanger loci) recovered the monophyly of Phrynidae and Phrynoidea with full support^10^. The success in retrieving these taxa only by the Bayesian CAT-GTR model may have been because site-heterogeneous mixture models better fit highly variable sequences and are less prone to systematic errors than classical empirical matrices^39–42^. In addition, PhyloBayes has been largely used in mitogenomics and phylogenomics of Arachnida to accommodate full across-site heterogeneity and account for possible sources of phylogenetic errors^13,22,43–45^. Moreover, the erroneous grouping of unrelated taxa in phylogenetic analyses is more prevalent when the taxon sampling is low and the taxa sampled are distantly related^13,41,46^, as in our data sets. Nevertheless, as previously mentioned, there were only two available mitogenomes of whip spiders, and even with our contribution of three new species, more than doubling that amount, we still have a tiny fraction of an order having around 270 species. Therefore, a global mitogenomic phylogenetic analysis of whip spiders is far from being reached with current datasets, and it is important to clarify that such an attempt falls outside the scope of this paper. Our focus is on providing new mitochondrial genomes of individual species and exploring the phylogenetic relationship of these taxa with relatives based on these new data.

## Conclusions

The mitogenomes of *Charinus* and *Heterophrynus* herein provided were compared with those of *Damon*, *Phrynus*, and other related arachnid orders, allowing us to find general features shared only or mainly by Amblypygi taxa. Some of these attributes are: (1) biases in nucleotide usage (Cs over Gs), (2) preservation of the presumed ancestral order of genes in arthropods, and (3) most of the tRNAs presenting the typical cloverleaf-shaped secondary structure. These shared features revealed the evolutionary trends followed by the most recent common ancestors of modern whip spiders. Other results were already expected, considering the nature of the mtDNA, as we observed Ka/Ks < 1 for all PCGs, a feature that is widespread in the mitogenomes of other arachnids. The phylogenetic analyses assigned the three species mitogenomes to their respective genera and confirmed *Charinus carajas* and *C*. *ferreus* as sister and well-delimited species. We highlight the supposed advantages of using the Bayesian CAT model to better account for rate heterogeneity among amino acid sites. In general, the results in this paper filled a knowledge gap in the genetics of whip spiders and provided data for future studies on the molecular biodiversity of Amblypygi, particularly those that benefit from the biological properties of the mtDNA, such as demographic histories and phylogeography. Such evolutionary studies will demand a denser sampling of individuals per species and would be especially important for *Charinus carajas* and *C*. *ferreus*, two short-range endemics and cave-dwelling species in the Amazon^5^, for which there is no genetic data assessing the status of their populations.

## Methods

### Specimen sample and DNA extraction

The amblypygids herein sequenced were collected in the caves located within the limits of the *Floresta Nacional de Carajás*, in the southeast state of Pará, Brazilian Amazon. All specimens were fixed in 95% ethanol and stored at − 20 °C until used for DNA extraction. Specimen collections were authorized by the environmental agency ICMBio/MMA under license number 49994-12. Genomic DNA of the samples was isolated using the DNeasy Blood & Tissue Kit (Qiagen), following the manufacturer’s protocol for insects, and stored at − 20 °C. Individual isolates from which the sequences were obtained are ITV2673 for *H. longicornis*; ITV32368, ITV32372, and ITV32381 for *C*. *ferreus*; and ITV32399, ITV32401, and ITV32410 for *C*. *carajas*.

### Genome sequencing, assembly, annotation, and analyses

Paired-end libraries were constructed using approximately 50 ng of DNA with the QXT SureSelect (Agilent Technologies) and sequenced in an Illumina platform with a NextSeq 500 v2.5 kit high-output (300 cycles), following the manufacturer’s instructions. Mitochondrial genomes were *de novo* assembled with NOVOPlasty v4.2^47^ using the *cox1* nucleotide sequence of *Charinus neocaledonicus* (GenBank: MT040916) as seed input, and annotations were performed with MITOS2^48^. MEGA X^49^ was used to calculate nucleotide composition, amino acid frequency, and relative synonymous codon usage (RSCU). The ratios of non-synonymous substitutions (Ka) per synonymous (Ks) substitutions of each protein-coding gene (PCGs) were estimated in the program DnaSP v6^50^ using all the available mitogenomes of whip spiders (Supplementary Table S1). Strand asymmetry was calculated by the following formulas: AT skew = (A% − T%)/(A% + T%) and GC skew = (G%−C%)/(G%+C%)^51^.

### Model selection and phylogenetic analyses

We conducted two distinct phylogenetic inferences. In one, we used 12 PCGs (all except *nad6*) and the two ribosomal RNAs (rRNAs) of our samples and from the mitogenomes of *Phrynus* sp., *Damon diadema*, *Mastigoproctus giganteus*, *Schizomus zhensis*, and *Trichonephila clavata*, available in the GenBank (Supplementary Table S1), to construct nucleotide (PCGs and rRNAs) and amino acid (AA alignments of PCGs) matrices. These matrices were narrow in terms of taxa sampled but broad in the number of characters used, with *T*. *clavata* used to root the trees. In the other inference, to include more amblypygid taxa, particularly those more closely related to *Charinus* and *Heterophrynus*, the nucleotide matrix of 14 genes was reduced to comprise only *cox1* and the two rRNAs genes (Supplementary Table S1). Resulting in a DNA matrix broad in the number of taxa used but narrow in terms of characters sampled, in which *M*. *giganteus* was used to root the trees.

Individual genes were independently aligned using MAFFT v7^52^ with the algorithm *Auto*. The alignments were trimmed to avoid a large amount of missing data at the ends and then concatenated into a unique matrix in Geneious Prime 2022.0.2 (https://www.geneious.com)^53^. For partitioning analyses, we defined data blocks based on genes and codon positions, one for each rRNA and one for each codon position of protein-coding genes, for the DNA alignments, while for the amino acid (AA) data set, we assigned data blocks based on genes. Hence, for the DNA data sets, we set five data blocks for the three-gene matrix and 35 for the 14-gene matrix (we joined *atp6* and *atp8* due to their contiguity in the mitogenomes), whereas for the AA data set, we set 12 data blocks.

We used ModelFinder^54^ to find best-fit partitioning schemes^55^ and model selection for the maximum-likelihood (ML) analyses according to BIC (Supplementary Table S2). We employed the edge-unlinked partition model that accounts for heterotachy, i.e., rate variation across lineages, whilst rate heterogeneity across sites (RHAS) was accommodated by the FreeRate model^56^ (-Q partitions.nex -m MF + MERGE). We used IQ-TREE 2 with hill-climbing and stochastic NNI as the tree search strategy^57^. Nodal support was estimated via 1000 ultrafast bootstrap (UFBoot) replicates and Shimodaira–Hasegawa aLRT (SH-aLRT) with 1000 replicates (-B 1000 -alrt 1000)^58^.

For the Bayesian analyses of the DNA data sets, we used MrBayes 3.2^59^ for the phylogenetic reconstructions and PartitionFinder2^60^ to find best-fit partitioning schemes and model selection as this program provides partition definitions in the specific MrBayes block format (Supplementary Table S3). In PartitionFinder2, branch lengths were set to ‘unlinked’ for all analyses and the partitioning scheme search was set to ‘all’ for the reduced three-gene matrix and ‘greedy’ for the complete data sets. For the trees search, we executed two independent runs of four chains, sampling generations until we reached good convergence and stationarity results (i.e., ESS > 200 in combined runs). Nonetheless, for the Bayesian phylogenetic reconstructions using the AA alignments, we utilized PhyloBayes MPI^61^. This software implements the CAT infinite mixture model for modeling among-site variation in the rate of substitution^62^. We initially implemented the CAT-GTR model (virtually always the better-fitted model among those implemented in PhyloBayes), where exchange rates are always considered free parameters and inferred from the data, and checked the runs for satisfactory convergence (i.e., maxdiff < 0.1)^61,62^.

## Electronic supplementary material

Below is the link to the electronic supplementary material.

Supplementary Material 1

## Data Availability

The complete mitogenomes herein sequenced were deposited in GenBank under the accession numbers OQ758255 (ITV32399), OQ758256 (ITV32401) and OQ758257 (ITV32410) for Charinus carajas; OQ108440 (ITV32368), OQ108441 (ITV32372) and OQ108442 (ITV32381) for C. ferreus; and OP573470 for Heterophrynus longicornis.
