# Supplementary material for: New mitochondrial genomes of three whip spider species from the Amazon (Arachnida, Amblypygi) with phylogenetic relationships and comparative analysis
Source: Sci Rep. 2024 Nov 1;14:26271. doi: 10.1038/s41598-024-77525-0 (PMC11530452; doi:10.1038/s41598-024-77525-0)
Supplement: Supplementary file 1 — Supplementary Material 1 [file 41598_2024_77525_MOESM1_ESM.docx]

Supplementary Information

**New mitochondrial genomes of three whip spider species from the Amazon (Arachnida, Amblypygi) with phylogenetic relationships and comparative analysis**

Acácio Freitas Nogueira, Eder S. Pires, Guilherme Oliveira, Leonardo Carreira Trevelin & Santelmo Vasconcelos

**Contents**

**Supplementary Figure S1**. The codon usage and relative synonymous codon usage (RSCU) of each amino acid in the mitogenomes of *Charinus* *carajas*, *C*. *ferreus*, and *Heterophrynus* *longicornis*.

**Supplementary Figure S2**. tRNA secondary structures of *Charinus carajas*.

**Supplementary Figure S3**. tRNA secondary structures of *Charinus* *ferreus*.

**Supplementary Figure S4**. tRNA secondary structures of *Heterophrynus longicornis*.

**Supplementary Table S1**. GenBank accession numbers of the previously available arachnid mitochondrial sequences used for comparisons and phylogenetic analyses.

**Supplementary Table S2.** Best-fit partitioning schemes and models per partition selected by ModelFinder for the data sets in the ML analyses.

**Supplementary Table S3.** Best-fit partitioning schemes and models per partition selected by PartitionFinder2 for the data sets in the Bayesian inferences.


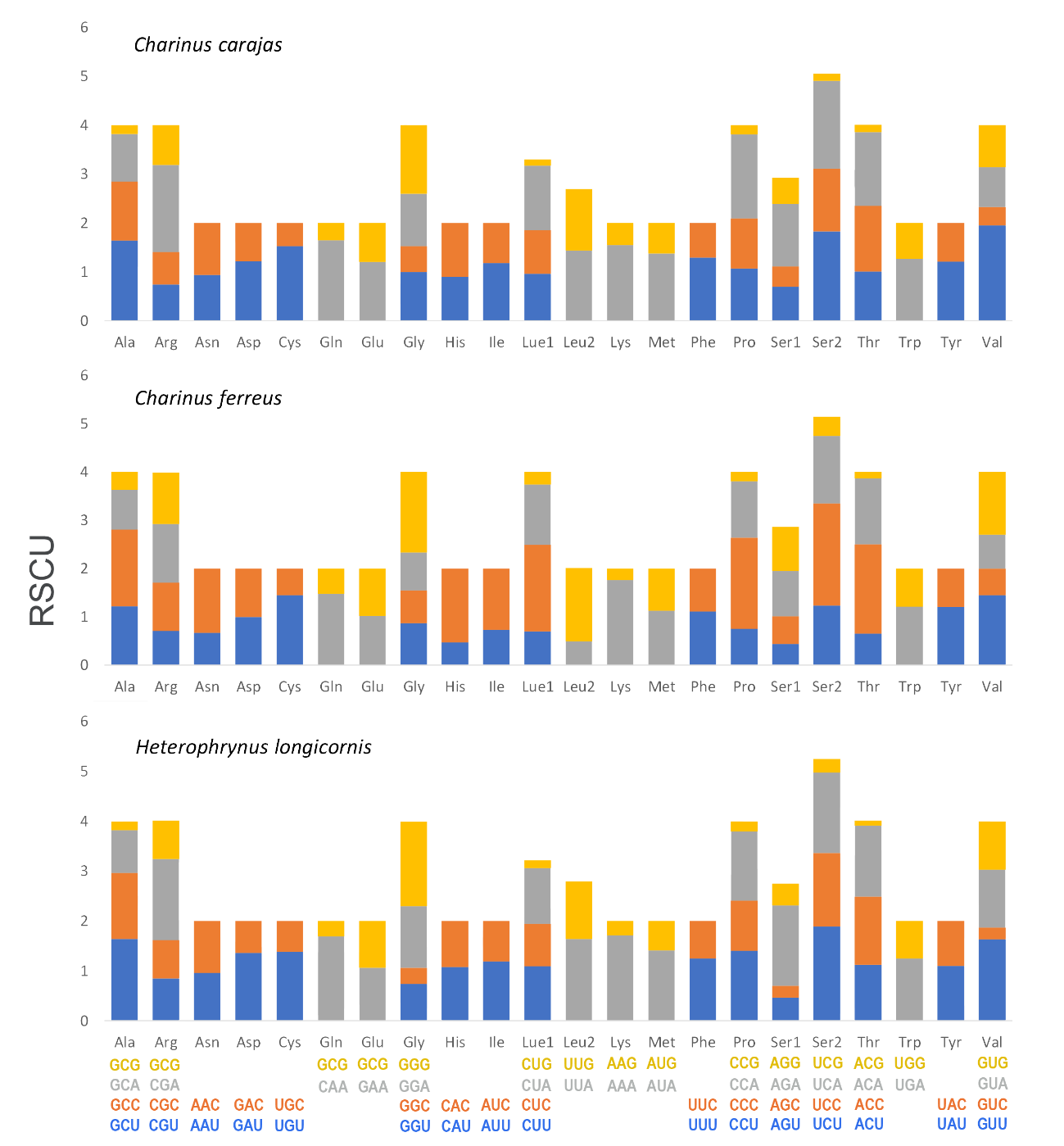


**Supplementary Figure S1**. The codon usage and relative synonymous codon usage (RSCU) of each amino acid in the mitogenomes of *Charinus* *carajas*, *C*. *ferreus*, and *Heterophrynus* *longicornis*. The code color in the horizontal axis corresponds to the same color in the columns. The statistics were computed in MEGA X.


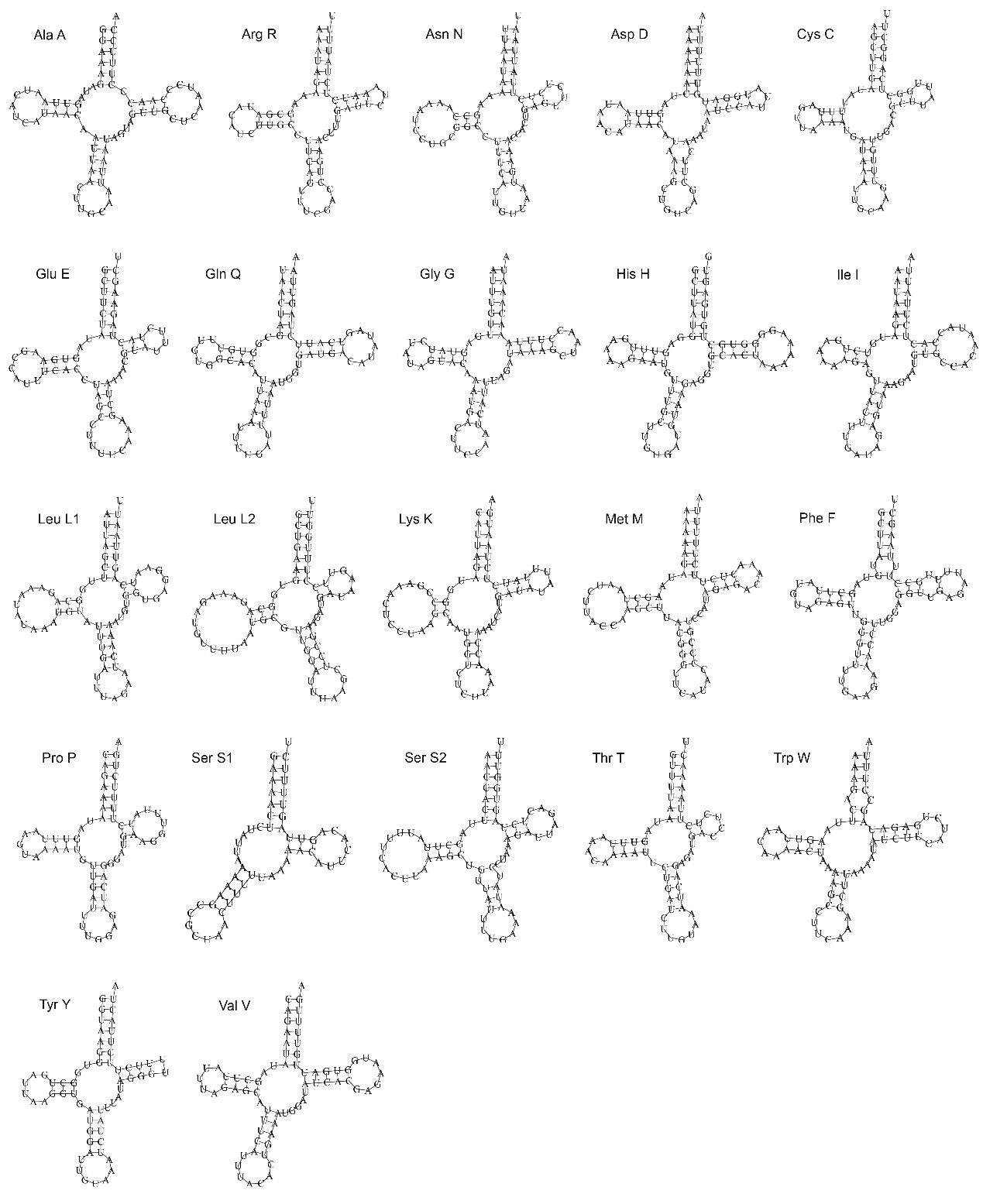


**Supplementary Figure S2**. tRNA secondary structures of *Charinus carajas*.

**
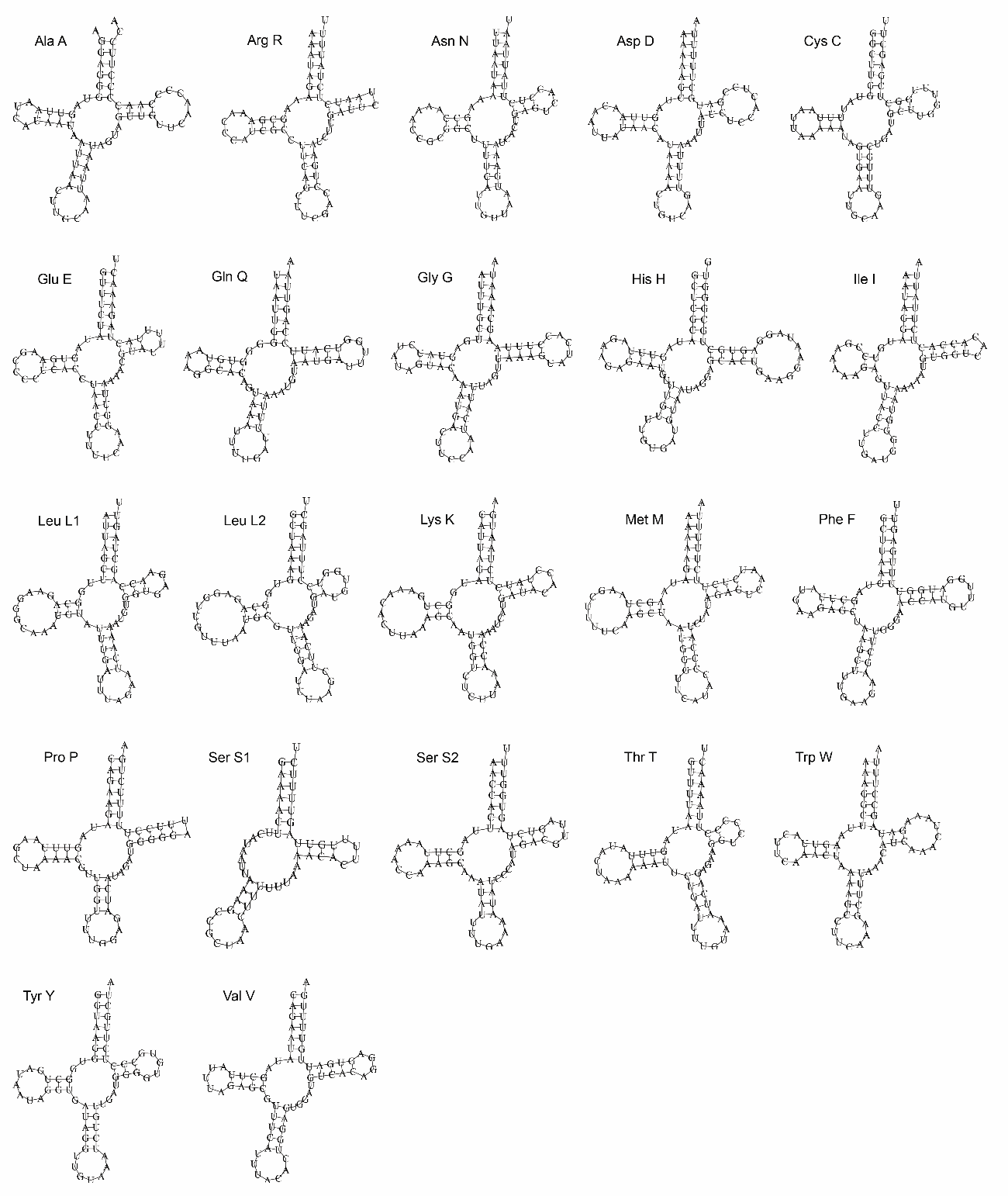
**

**Supplementary Figure S3**. tRNA secondary structures of *Charinus* *ferreus*.

**
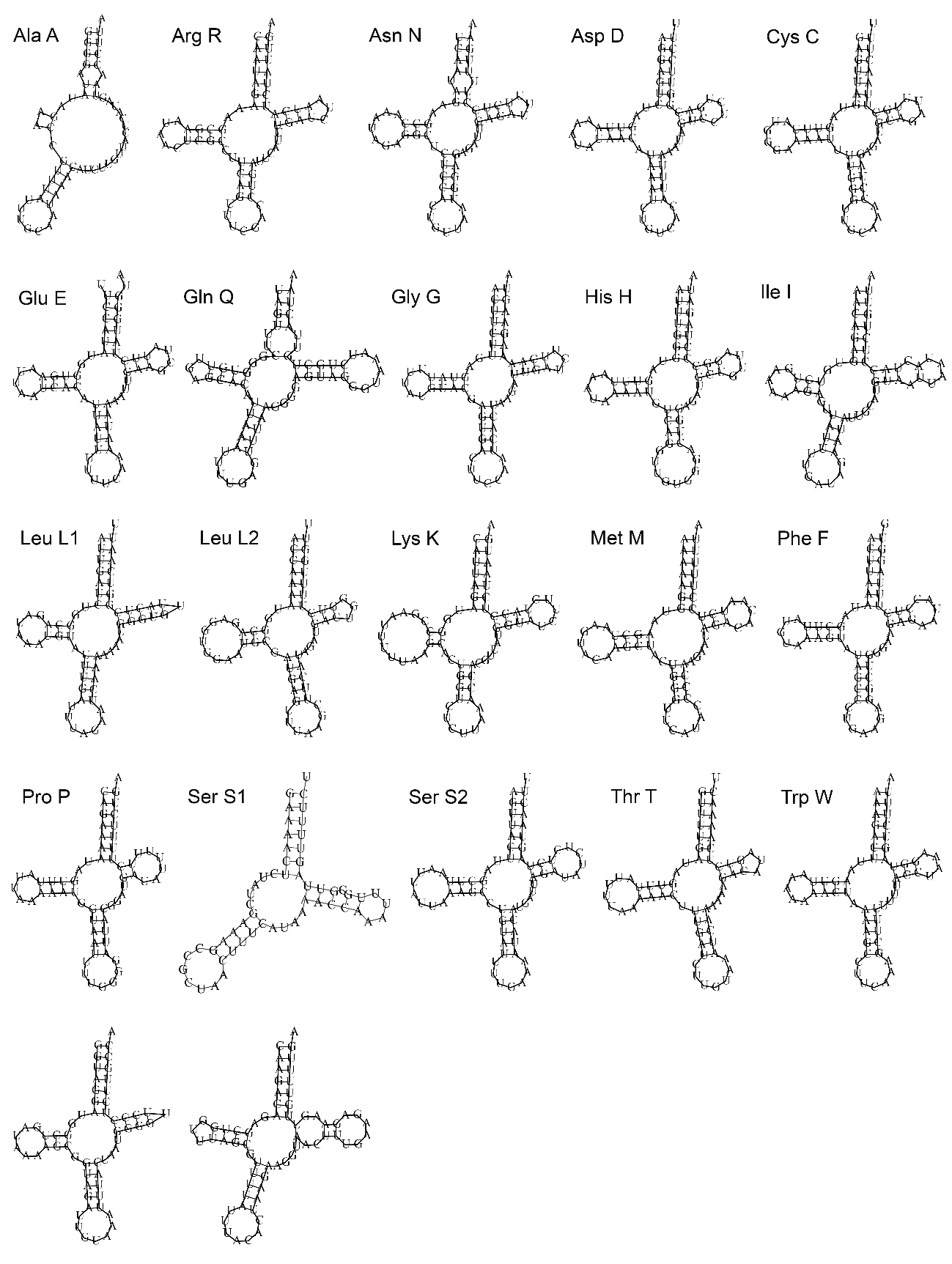
**

**Supplementary Figure S4**. tRNA secondary structures of *Heterophrynus longicornis*.

**Supplementary Table S1**. GenBank accession numbers of the previously available arachnid mitochondrial sequences used for comparisons and phylogenetic analyses.

| Taxon | Accession no. | | | |
| --- | --- | --- | --- | --- |
|  | complete mitogenome | *cox1* | 12S rRNA | 16S rRNA |
| *Damon diadema* | FJ204233 | AY829947 | AY829867 | AY829886 |
| *Phrynus sp.* | EU520641 |  |  |  |
| *Mastigoproctus* *giganteus* | EU520643 |  |  |  |
| *Schizomus zhensis* | OL544939 |  |  |  |
| *Cheiracanthium triviale* | MN334527 |  |  |  |
| *Nephila* *pilipes* | MW178204 |  |  |  |
| *Trichonephila* *antipodiana* | MW178205 |  |  |  |
| *Trichonephila* *vitiana* | MW178206 |  |  |  |
| *Trichonephila* *clavata* | NC_008063 |  |  |  |
| *Acanthophrynus coronatus* |  | MW410161 | MW411571 | MW411542 |
| *Paraphrynus robustus* |  | MT738756 | MT753022 | MT734766 |
| *Phrynus longipes* |  | MT040911 | MF806134 | MF806089 |
| *Phrynus marginemaculatus* |  | MW410164 | MW411574 | MW411545 |
| *Heterophrynus alces* |  | MT899148 |  |  |
| *Heterophrynus batesii* UFMG15426 |  | MT899141 |  |  |
| *Heterophrynus longicornis* LP 3830 |  | MW410162 | MW411572 | MW411543 |
| *Heterophrynus longicornis* Lo55 |  | MT899094 |  |  |
| *Musicodamon atlanteus* |  |  | AY829863 | AY829882 |
| *Charinus africanus* |  | MT040897 | MF806121 | MF806078 |
| *Charinus australianus* |  | MT040907 | MF806130 | MF806086 |
| *Charinus aguayoi* |  | MT040938 | MF806163 | MF806112 |
| *Charinus dominicanus* |  | MT040906 | MF806129 |  |
| *Charinus gertschi* |  | MT040903 | MF806127 | MF806084 |
| *Charinus pescotti* |  | MT040920 | MF806145 | MF806099 |
| *Charinus potiguar* |  | MT040921 | MF806146 |  |
| *Charinus reddelli* |  | MT040922 | MF806147 | MF806100 |
| *Charinus rocamadre* |  | MK801767 |  | MK810724 |
| *Charinus sillami* |  | MT040929 | MF806154 |  |
| *Charinus taboa* |  | MT040934 | MF806159 |  |
| *Charinus vulgaris* |  | MT040939 | MF806164 | MF806113 |
| *Sarax rimosus* |  | MT040923 | MF806148 | MF806101 |
| *Sarax singaporae* |  | MT040931 | MF806156 | MF806107 |
| *Sarax yayukae* |  | MT040940 | MF806165 | MF806114 |
| *Weygoldtia davidovi* |  | MT040896 | MF806128 | MF806085 |

**Supplementary Table S2.** Best-fit partitioning schemes and models per partition selected by ModelFinder for the data sets in the ML analyses. The suffixes p1, p2, and p3 indicate the first, second, and third codon positions of the PCGs, respectively.

| **Data sets** | **Models** | **Partitions** |
| --- | --- | --- |
| 3 mtDNA genes | TIM3+F+I+G4 | *rrnS,* *rrnL* |
|  | TNe+I+G4 | *cox1*-p1, *cox1*-p2 |
|  | TIM2+F+R5 | *cox1*-p3 |
| 14 mtDNA genes | TPM3u+F+I+G4 | *rrnS, rrnL* |
|  | GTR+F+R2 | *atp*-p, *atp*-p2, *atp*-p3, *nad2*-p1, *nad3*-p1, *nad3*-p3, *nad4l*-p1 |
|  | TPM2u+F+I+G4 | *cob*-p1, *cox2*-p1, *cox3*-p1, *nad2*-p2 |
|  | TIM3+F+G4 | *cob*-p2, *cox1*-p1, *cox1*-p2, *cox2*-p2, *cox3*-p2 |
|  | HKY+F+G4 | *cob*-p3, *nad2*-p3 |
|  | HKY+F+I+R2 | *cox1*-p3, *cox2*-p3, *cox3*-p3 |
|  | TIM+F+I+G4 | *nad1*-p1, *nad4*-p2, *nad5*-p1, *nad5*-p2 |
|  | GTR+F+G4 | *nad1*-p2, *nad3*-p2, *nad4*-p3, *nad4l*-p2 |
|  | HKY+F+R2 | *nad1*-p3 |
|  | HKY+F+G4 | *nad4*-p1, *nad4l*-p3, *nad5*-p3 |
| 12 PCG (AA) | mtMet+G4 | *atp6*, *nad3* |
|  | mtMet+R2 | *atp8* |
|  | mtART+R3 | *cob*, *cox1*, *cox2*, *cox3* |
|  | mtInv+F+I | *nad1*, *nad4*, *nad5* |
|  | mtInv+G4 | *nad2*, *nad4l* |

**Supplementary Table S3.** Best-fit partitioning schemes and models per partition selected by PartitionFinder2 for the data sets in the Bayesian inferences. The suffixes p1, p2, and p3 indicate the first, second, and third codon positions of the PCGs, respectively.

| **Data sets** | **Models** | **Partitions** |
| --- | --- | --- |
| 3 mtDNA genes | GTR+I+G | *rrnS,* *rrnL* |
|  | SYM+I+G | *cox1*-p1, *cox1*-p2 |
|  | HKY+G | *cox1*-p3 |
| 14 mtDNA genes | GTR+I+G | *rrnS* ,  *rrnL* , *nad4l*-p1, *nad4*-p2, *nad1*-p1, *nad5*-p1 |
|  | GTR+G | *nad3*-p2, *atp*-p1, *nad2*-p2 |
|  | HKY+I+G | *cox2*-p3, *cox1*-p3, *cob*-p3, *nad3*-p3, *cox3*-p3, *nad2*-p3, *atp*-p2 |
|  | GTR+G | *nad2*-p1, *nad3*-p1, *atp*-p3 |
|  | GTR+G | *cox1-*p1*, cob*-p1*, cox2*-p1*, cox3*-p1 |
|  | GTR+G | *cox1*-p2*, cob*-p2*, cox2*-p2*, cox3*-p2 |
|  | GTR+G | *nad4l*-p2*, nad5*-p2*, nad1*-p2*, nad4*-p3 |
|  | HKY+G | *nad1*-p3, *nad4l*-p3, *nad4*-p1, *nad5*-p3 |
